# Development of Health Parameter Model for Risk Prediction of CVD Using SVM

**DOI:** 10.1155/2016/3016245

**Published:** 2016-08-09

**Authors:** P. Unnikrishnan, D. K. Kumar, S. Poosapadi Arjunan, H. Kumar, P. Mitchell, R. Kawasaki

**Affiliations:** ^1^Biosignals Lab, School of Electrical and Computer Engineering, RMIT University, Melbourne, VIC 3001, Australia; ^2^Eastern Health, Melbourne, VIC 3128, Australia; ^3^Centre for Vision Research, Department of Ophthalmology, Westmead Millennium Institute, University of Sydney, Sydney, NSW 2006, Australia; ^4^Department of Public Health, Yamagata University Faculty of Medicine, Yamagata 990-9585, Japan

## Abstract

Current methods of cardiovascular risk assessment are performed using health factors which are often based on the Framingham study. However, these methods have significant limitations due to their poor sensitivity and specificity. We have compared the parameters from the Framingham equation with linear regression analysis to establish the effect of training of the model for the local database. Support vector machine was used to determine the effectiveness of machine learning approach with the Framingham health parameters for risk assessment of cardiovascular disease (CVD). The result shows that while linear model trained using local database was an improvement on Framingham model, SVM based risk assessment model had high sensitivity and specificity of prediction of CVD. This indicates that using the health parameters identified using Framingham study, machine learning approach overcomes the low sensitivity and specificity of Framingham model.

## 1. Introduction 

Cardiovascular disease (CVD) is the single biggest cause of mortality worldwide [1]. Globally, an estimated 17.5 million deaths were attributable to CVD in 2005 [2, 3]. Early identification of persons with higher risk of CVD is useful for timely implementation of preventative strategies for preventing cardiac episodes that lead to death or disabilities [3, 4]. For this purpose, risk factors for CVD [4] such as cholesterol, hypertension, and diabetes have been identified and various risk assessment models and techniques have been developed [5].

The commonly used risk assessment models for CVD prediction are the Framingham Risk Score [1], Reynolds Risk Score [6], QRISK [7], Prospective Cardiovascular Munster Heart Study (PROCAM) [8], the Systematic COronary Risk Evaluation (SCORE) system [9], and UKPDS [10]. Many of these have been adapted in primary care as simplified charts, tables, computer programs, and web-based tools which are routinely referred to in policy documents and guidelines.

The accuracy of the Framingham Risk Score is superior to any single risk factor. However its predictive power leaves room for improvement because the sensitivity and specificity are not very high [11–14]. It has been observed that the overall absolute coronary risk assigned to individuals in the United Kingdom has been significantly overestimated [11]. This highlights the necessity to refine the prediction models.

There can be a number of reasons underpinning the low prediction of CVD risk when using Framingham equation. This research has studied two of the possible causes for poor sensitivity and specificity of the equation, difference in demographics, and the linearity assumptions of the model. One cause for poor sensitivity and specificity could be due to the difference in the demographics of the population being studied compared with the Framingham population [4] which was used to develop the Framingham equation. The issue of demographics was showcased in a 2015 study which reported that the magnitude of the effect of different cardiovascular risk factors upon a patient is highly dependent on their ethnicity [15]. If this is the reason, it would require that the equation parameters (model) would have to be redefined for different demographics. Redefining the parameters may not always be possible because the modelling requires large amount of longitudinal data, which may not be available outside major hospital centres. However, identifying the cause of differences between groups can lead to better understanding and also provide the reason for developing new databases.

Another reason for poor performance can be attributed to the type of model. Framingham equation and other similar techniques are generalized linear equations. However, the relationship between the multiple factors associated with the health of large number of people may require more complex representation and is not suitable for linear approximation. To overcome this problem, the redevelopment of the model is required without the constraints of linearity.

This work has tested whether population difference or model type is the cause of poor outcomes for Framingham model. This has been done by developing risk assessment models using a longitudinal population database that compares the specific linear equation and machine learning methods. The commonly accepted health parameters that have been described by Framingham model were used and the scope of this study was to compare machine learning technique, linear regression, and direct use of Framingham model for identification of these parameters with disease. The linear equation was used to test the effect of customisation by using coefficients obtained using local database which would improve the results. To determine if the parameters used by Framingham model are relevant to a different database, this study measured the sensitivity and specificity obtained using support vector machine (SVM). While machine learning is generally expected to provide improved results, this study tested the effect of parameters used in the Framingham model which are relevant to a different database.

## 2. Materials and Methods

### 2.1. Database

To ensure that the study had adequate power, a large longitudinal database is required. Such population databases provide the natural numbers of cases and controls that are matched as in the real world. The longitudinal database ensures the population baseline for demographics and ethnicity.

In this study, the Blue Mountain Eye Study (BMES) [16] database was used. This database was created from a population based cohort study which recorded eye and other health outcomes in an urban Australian population greater than 49 years of age. The majority of this population (~99%) was of European descent. Baseline participants (*n* = 3654) represented 82.4% of those eligible in the selected postcode areas. The population group had a 5-year follow-up protocol with the last examination conducted 15 years after baseline examination. Participants of the study provided written informed consent prior to their involvement and any data collection.

The study population was followed up at 5-year intervals and the latest follow-up examination was conducted 15 years since the baseline examination. The study was approved by the Western Sydney Area Health Service Human Research Ethics Committee. Written informed consent was obtained from all participants prior to recording their data.

The 5- and 10-year follow-up data was used in this study. The database consisted of health and other parameters that have been identified by Framingham study [1] and consisted of gender, smoking status, cholesterol (combined and high-density), systolic and diastolic blood pressure, body mass index, diabetes, and hypertension. These have been described in detail in Table 1.

People who had CVD episodes before the baseline examination or who died during the follow-up period due to a noncardiovascular aetiology were excluded from the study. The size of the study became 2770 subjects after the above exclusions. After a further 364 patients were excluded due to missing data, the remaining database of 2406 people had 1450 females and 956 males. The CVD cases were divided in two: hard and soft. Incident “hard CVD” included myocardial infarction, stroke, bypass surgery for coronary artery disease (CAD), or death from CAD. Self-reported angina was categorized as a “soft CVD” incident outcome. The mortality data were obtained by linkage with the Australian National death Index (NDI) and all nonexact matches were manually analyzed and accepted only if the mismatch was a single noncritical characteristic. In this set, there were 535 (267 women and 268 men) who had incident CVD (hard and soft) events in a period greater than 5 years but less than 10 years and this is shown in Table 1.

### 2.2. Data Management

The data was randomly divided into two subsets corresponding to training data and test data using Scikit [21]. The training data consisted of 1896 (approximately 80%) and the balance of 510 samples (approximately 20%) of the total data was for testing. Thus, 80% of the data was used for training and the balance of 20% for testing purposes, with no overlap. This data is available online and in accordance with privacy regulations.

Pattern recognition and risk prediction techniques applied to population health data may suffer when these datasets are highly imbalanced. To overcome this imbalance, Synthetic Minority Oversampling Technique (SMOTE) [22] was used to boost the minority class (CVD case) numbers by 400% in the training data by artificially generating samples using a nearest neighbour approach [23].

### 2.3. Framingham Risk Equation

The Framingham model provides a gender-specific model for various cardiovascular outcomes and is the basis for estimating cardiovascular risk profile and number of major public health policies [24]. We used a 10-year general cardiovascular risk prediction Framingham equation (FEq) for our analysis [1] with the regression coefficients and hazard ratios shown in Table 2.

The outcome of the equation is a risk of CVD over the following 10 years. It was applied to data on each subject in the test database (described in Data Management) and a risk percentage obtained. These predictions were compared with the known CVD episodes from the records. To interpret the risk percentage obtained with the information of the CVD episodes, weighted statistical analysis was performed to optimally classify the cases and controls using the training data.

For the training data, this threshold was found to be 22.3%, and this was used on the test data to separate the case and control. According to the parameters in FEq, the samples that were above the age of 79 were “not classifiable.”

### 2.4. Logistic Regression Analysis (LRA)

LRA develops a linear equation to best model a database with multiple features and two outcomes. Linear regression is performed to maximize the separation between the two outcomes. Consider that there are *p* samples in the database that belong to two classes, and there are *n* features (predictors). With the two classes, (i) CVD and (ii) no-CVD, logistic regression using the probability function was used to determine the relationship between the predictors. This was based on the conditional probability and described in the equation below:

(1) $$\begin{array}{c} P\left(\;CVD\;|\;X\;\right)=\frac{e^{\beta_{0}+\beta_{1}x_{1}+\cdots +\beta_{p}x_{pn}}}{1+e^{\beta_{0}+\beta_{1}x_{1}+\cdots +\beta_{p}x_{n}}}. \end{array}$$

In this equation, the probability of CVD based on the predictor vector *X* is obtained by considering each predictor, *x*, and *β* is the regression coefficient which indicates the relevance of the predictor or the contribution of the predictor on the outcome class. LRA was trained to obtain the parameters of each feature using the training section and tested using the test section of data (as described in Data Management). The default value *P*(CVD∣*X*) > 0.5 was used for classification. The prediction was performed on the test data (*n* = 510 subjects) and compared with prior knowledge of the CVD episodes. The weaknesses of Framingham equation with 79 years being the limit of the age and having predefined coefficients have been overcome by LRA.

### 2.5. Support Vector Machine (SVM)

SVM is a set of related supervised learning methods that are used for prediction and regression analysis with applications in fields such as clinical and population based data [25], text classification, bioinformatics, handwriting recognition, and image analysis. These have to be trained using examples and do not require the user to define the relationship between the various factors. They are suitable for situations where appropriate and representative examples of all the different categories (classes) are available. SVM have the advantage that these do not require linear relationships or independence between the input features and thus are more suitable for clinical data classification.

As a first step, the SVM was trained using the training subset (refer to Data Management) which was used as the input to the SVM and the target output was the known history of CVD episodes (as defined earlier) during the 5 to 10 years after time zero. The parameters for the SVM,* Kernel*, *C*, and *γ* were identified using grid search method reported by Bergstra and Bengio [26]. This method [26] exhaustively generates possible values from a grid of the following specified two parameter values:

(i) first with linear kernel and *C* values in (1, 10, 100, 1000),
(ii) the second one with an RBF kernel and the cross product of *C* values ranging in (1, 10, 100, 1000) and gamma values in (0.001, 0.0001).

All possible combinations of parameter values were fitted on the dataset and evaluated with an output score. Based on the score the following parameter values were used in this study:

(i) Radial Basis Function (RBF) Kernel,
(ii) *C* = 100,
(iii) *γ* = 0.01.

This SVM model was used to rank the parameters in terms of their relevance based on the weights obtained during the training (Table 3) [20]. The trained SVM was tested using the subsample of the test dataset (510 samples). This strategy ensured that the test data was independent of the training data. Diagnostic odds ratios were calculated [17] to compare its performance with the Framingham model and logistic regression analysis.

## 3. Results

Table 3 shows the relevance of the features as obtained from the ranking of logistic regression coefficients obtained for BMES dataset, while Table 4 reports the ranking of these features based on SVM weights. Comparing the results from Tables 2 [Table tab3] [Table tab4]–5, it is observed that the highest three relevant factors (features) are the same for the three methods [1]: age, BMI, and current smoker.

A confusion matrix shows the extent of the mislabelling performed by the prediction algorithm. Tables 5 [Table tab6]–7 show the confusion matrices for FEq, LRA, and SVM, respectively. Each row represents the instances in a predicted class, while each column represents the instances in an actual class. From these results, it is observed that the correct prediction using FEq was 40, using LRA was 50, and using SVM was 71 from a total of 104 CVD cases.

The confusion matrices also show that the number of false positives when the prediction was performed using Framingham was 108, using SVM was 57, and using LRA was 68. The results also show that the number of cases that were falsely identified to be controls by FEq were 37, 54 by LRA, and 33 by SVM. However, while SVM and LRA classified all the test samples (104 cases and 406 controls), there were 27 cases and 46 controls that were unclassifiable by FEq because of the age of these people being above 79 years. This is a major limitation for FEq, especially when we have an ageing population with significant population being older than 79 years.

**Figure 1:**
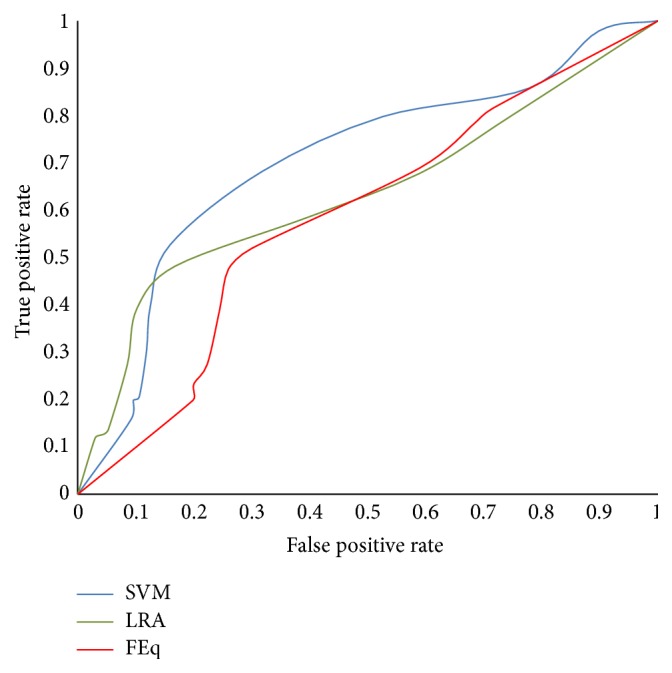
ROC graph for SVM, LRA, and FEq.

The sensitivity and specificity obtained from SVM analysis, logistic regression, and FEq are shown in Table 8. This table also lists the range for 95% confidence interval (CI) of the data. Sensitivity obtained from the FEq was 0.52 (95% CI: 0.4096 to 0.6275), from the LRA was 0.48 (95% CI: 0.3817 to 0.5809), and from the SVM was 0.682 (95% CI: 0.589 to 0.764). This shows that the sensitivity of the FEq and logistic analysis is comparable, while that of SVM is better and thus provides better risk assessment. This is also confirmed with the ROC analysis curve as shown in Figure 1 and it is also observed from the area under ROC curve (AUC) corresponding to SVM which has the highest coverage (Table 8).

From Table 8, it is observed that specificity of the SVM classifier (0.859) was the highest when compared with FEq (0.70) and LRA (0.832). It is also observed that the diagnostic odds ratio was significantly higher for SVM (13.17) when compared with FEq (2.52) and LRA (4.602) and indicates that SVM is more effective in the diagnostic test. The AUC test shows that the SVM results were greatly improved (0.71) compared with Framingham (0.57) or LRA (0.63).

The statistical significance test between the sensitivity and specificity of prediction was performed by comparing the AUC measured from the ROC curves for SVM, LRA, and FEq [18, 19]. When comparing the SVM technique with LRA and FEq, there were significant differences between SVM and FEq (*P* < 0.0002) and also LRA (*P* < 0.02).

## 4. Discussion

These findings show that there are a large number of unclassifiable cases and controls when using Framingham equation (FEq) due to the age constraints of the equation and in this database, 27 cases corresponding to ~26% of all cases were not classifiable. This is a major weakness because with our ageing society significant amount of the population is older than 79 years. The results show that only 40 out of total 104 cases were identified correctly. LRA classified all samples and 50 of the 104 cases were identified correctly and SVM identified 71 cases correctly. This shows that while LRA overcame some of the limitations, it was not sufficient and the labelling of the outcome lacked sensitivity and specificity.

The results also showed that that there were a large number of false positives by FEq and 108 out of total of 406, or approximately 27% of the controls were misclassified to be case. This number reduced to 68 (~17%) when the LRA was used and 57 (~14%) when the SVM was used. The diagnostic odds ratio for FEq is 2.52, LRA is 3.05, and SVM is 13.17. SVM gave the highest correct predictions, lowest false positives, and false negatives and classified all the samples.

This study has shown that machine learning approach gave significantly better AUC. The study also demonstrated that the health parameters identified using Framingham model are relevant for other populations such as Blue Mountains in Australia, but when the weaknesses of the earlier model are overcome using machine learning approach, it should be noted that in this study SVM is an example of machine learning classifiers and was selected as an example to demonstrate the effectiveness of using machine learning based health parameter classification.

## 5. Conclusions

This study has compared the linear model and SVM approaches to classify the health features that are used by Framingham equation. To ensure that there is no bias due to differences in the database, all the analyses were performed on one database, BMES, which is a population based database that is well regarded for quality, duration, and size [14].

LRA and FEq are based on linearity assumption. However the FEq parameters were determined historically using Framingham database while LRA was trained on the local database to classify all subjects irrespective of the age. This would explain why LRA had improved true positive prediction of CVD (50 compared with 40), but there was also an increase in the false negatives (54 compared with 37 for FEq). Overall, the SVM performed significantly better. This may be attributed to SVM not being restricted by linearity which allows for nonlinear separation between the case and control class. It may also be based on the database being local. In conclusion, we propose that using an SVM with a local database may provide improved risk assessment. However, this needs to be tested on more databases and with more health parameters. It is also important to note that this work has only used the health parameters that were identified in Framingham study. However, it is now established that there are a number of other relevant parameters that need to be considered. Thus, it is essential that new databases with all the health parameters be developed and classified using SVM.

Support vector machine and other similar machine learning approaches are very useful in providing the flexibility that is lacking in linear models. However, there is the shortcoming that such an approach is a black-box approach and it is essential that training data should be balanced and representative of the complete database. There are also the difficulties for data points that may appear as outliers. This is often difficult to control and erroneous training can lead to incorrect outcomes. Thus, it is essential for the test results to be monitored by the experts. It is also important for the software to automatically identify the outliers which would trigger supervised assessment.

## Figures and Tables

**Table 1:** 10-year risk of CVD in the Blue Mountains Eye Study (BMES) for the 10 parameters.

Factor	Persons developed CVD in 10-year follow-up	Persons without CVD in 10-year follow-up
Gender M (F)	268 (267)	688 (1183)
Current smoker (past smoker or nonsmoker)	87 (448)	241 (1630)
Total cholesterol (high > 13.2/borderline (11–13.2)/normal < 11) (mmol/L) [17]	218/197/120	752/775/344
High-density lipoprotein cholesterol level (high > 3.3/borderline 2.2–3.3/low < 2.2) (mmol/L) [17]	154/170/211	659/665/547
Systolic blood pressure (high > 120/normal 90–120/low < 90) (mmHg) [18]	345/190/0	941/930/0
Diastolic blood pressure (high > 80/normal 60–80/low < 60) (mmHg) [18]	97/435/3	316/1554/1
Body mass index (low < 18.5/normal 18.5–24.9/high > 25) (kg/m^2^) [19]	10/222/303	33/766/1072
Diabetes (yes/no)	51/484	113/1758
Medication for hypertension (yes/no)	196/339	526/1345

**Table 2:** Coefficients in the Framingham risk estimation for 10-year general cardiovascular disease risk [1].

	Men^*∗*^ (10-year baseline survival: So(10) = 0.88431)				Women^*∗*^ (10-year baseline survival: So(10) = 0.94833)			
	Beta^*∗∗*^	*P* value	Hazard ratio	95% CI	Beta^*∗∗*^	*P* value	Hazard ratio	95% CI
Log of age	3.11296	<0.0001	22.49	(14.80, 34.16)	2.72107	<0.0001	15.20	(8.59, 26.87)
Log of body mass index	0.79277	<0.0066	2.21	(1.25, 3.91)	0.51125	<0.0609	1.67	(0.98, 2.85)
Log of SBP if not treated	1.85508	<0.0001	6.39	(3.61, 11.33)	2.81291	<0.0001	16.66	(8.27, 33.54)
Log of SBP if treated	1.92672	<0.0001	6.87	(3.90, 12.08)	2.88267	<0.0001	17.86	(8.97, 35.57)
Smoking	0.70953	<0.0001	2.03	(1.75, 2.37)	0.61868	<0.0001	1.86	(1.53, 2.25)
Diabetes	0.53160	<0.0001	1.70	(1.37, 2.11)	0.77763	<0.0001	2.18	(1.63, 2.91)

^*∗*^The 10-year risk for women can be calculated as 1 − 0.94833^exp⁡(Σ*βX*−26.0145)^, where *β* is the regression coefficient and *X* is the level for each risk factor; the risk for men is given as 1 − 0.88431^exp⁡(Σ*βX*−23.9388)^.

^*∗∗*^Estimated regression coefficient.

**Table 3 tab3:** Potential risk features ranked by weights obtained using support vector machine (SVM) feature selection [20], Blue Mountains Eye Study 10-year follow-up data.

Rank	Attribute	SVM weight
1	Age (per 1 year)	3.21660913
2	Body mass index (per 1 kg/m^2^)	0.15610062
3	Current smoker (past/never smoked)	0.06839195
4	Gender (male/female)	0.05784681
5	Total cholesterol (per 1 mmol/L)	0.04203396
6	Systolic blood pressure (per 1 mmHg)	0.01872727
7	High-density lipoprotein cholesterol (per 1 mmol/L)	0.01231242
8	Diabetes (versus no diabetes)	0.00610169
9	Medication for hypertension (versus no medication for hypertension)	0.00104436
10	Retinopathy (yes/no)	0.00064500
11	Diastolic blood pressure (per 1 mmHg)	0.00023068

**Table 4 tab4:** Regression coefficients and associated statistics obtained from BMES dataset for male and female subjects.

Feature	Male				Female			
	Coefficient	*ρ*	Odds ratio	95% confidence interval	Coefficient	*ρ*	Odds ratio	95% confidence interval
Age (per 1 year)	0.0144	<0.00001	1.015	(1.012, 1.017)	0.0110	<0.00001	1.011	(1.009, 1.012)
Body mass index (per 1 kg/m^2^)	0.0084	0.0024	1.008	(1.002, 1.013)	0.0018	0.2317	1.002	(0.998, 1.004)
Current smoker (versus past or never smoker)	0.0911	0.0005	1.095	(1.042, 1.153)	0.0749	0.0003	1.078	(1.034, 1.122)
Systolic blood pressure (per 1 mmHg)	0.0003	0.5700	1.000	(0.999, 1.001)	0.0003	0.3416	1.000	(0.999, 1.0009)
Medication for hypertension (versus no medication for hypertension)	−0.0042	0.8589	0.995	(0.953, 1.039)	0.0218	0.1521	1.022	(0.992, 1.0219)
Diabetes (versus no diabetes)	0.0460	0.1834	1.047	(0.979, 1.119)	0.0080	0.7852	1.008	(0.951, 1.067)
Total cholesterol (per 1 mmol/L)	0.0131	0.1615	1.013	(0.995, 1.032)	0.0016	0.8052	1.002	(0.989, 1.014)
High-density lipoprotein cholesterol (per 1 mmol/L)	0.0202	0.4524	1.020	(0.969, 1.074)	0.0046	0.7828	1.005	(0.972, 1.037)

Logistic regression constant *β*_0_ for male = −5.70203; logistic regression constant *β*_0_ for female = −5.30218.

**Table 5:** Confusion matrix using Framingham equation (FEq).

	Test negative	Test positive	Not classifiable	Total
No cardiovascular disease	252	108	46	406
Cardiovascular disease	37	40	27	104

*Total*	*289*	*148*	*73*	*510*

**Table 6 tab6:** Confusion matrix using logistic regression analysis (LRA).

	Test negative	Test positive	Not classifiable	Total
No cardiovascular disease	338	68	0	406
Cardiovascular disease	54	50	0	104

*Total*	*392*	*118*	*0*	*510*

**Table 7:** Confusion matrix using support vector machine (SVM).

	Test negative	Test positive	Not classifiable	Total
No cardiovascular disease	349	57	0	382
Cardiovascular disease	33	71	0	128

*Total*	*382*	*128*	*0*	*510*

**Table 8:** Sensitivity and specificity for SVM, Framingham model, and logistic regression model with diagnostic odds ratio.

Parameter	Model based on SVM classifiers		Framingham risk model		LRA model	
	Value	95% CI	Value	95% CI	Value	95% CI
Sensitivity	0.682	0.589 to 0.764	0.52	0.4096 to 0.6275	0.48	0.3817 to 0.5809
Specificity	0.859	0.8224 to 0.89	0.70	0.6508 to 0.745	0.83	0.7926 to 0.8675
Positive likelihood ratio	4.863	3.697 to 6.396	1.73	1.326 to 2.261	2.87	2.14 to 3.85
Negative likelihood ratio	0.369	0.278 to 0.491	0.69	0.539 to 0.871	0.62	0.52 to 0.75
Diagnostic odds ratio	13.173	7.999 to 21.696	2.523	1.529 to 4.162	4.602	2.892 to 7.324
AUC	0.71		0.57		0.63	
